# Serum sodium concentration and the progression of established chronic kidney disease

**DOI:** 10.1007/s40620-018-0541-z

**Published:** 2018-10-16

**Authors:** Nicholas I. Cole, Rebecca J. Suckling, Vipula Desilva, Feng J. He, Graham A. MacGregor, Pauline A. Swift

**Affiliations:** 1 South West Thames Renal Unit, Epsom and St Helier University Hospitals NHS Trust, Surrey, London, SM5 1AA UK; 2 Wolfson Institute of Preventative Medicine, Queen Mary University of London, London, UK

**Keywords:** Serum sodium, Serum potassium, CKD progression, eGFR decline

## Abstract

**Background:**

Higher serum sodium concentration has been reported to be a risk factor for the development of incident chronic kidney disease (CKD), but its relationship with the progression of established CKD has not been investigated. We hypothesised that increased serum sodium concentration is a risk factor for estimated glomerular filtration rate (eGFR) decline in CKD.

**Methods:**

This was a retrospective cohort study using data collected over a 6-year period, with baseline data obtained during the first 2 years. We included patients known to our renal service who had had a minimum of three blood tests every 2 years and an eGFR of < 60 mL/min/1.73 m^2^ at baseline. Exclusion criteria were renal replacement therapy, diabetes mellitus, heart failure and decompensated liver disease. A multiple linear regression model investigated the relationship between baseline serum sodium and eGFR decline after adjustment for confounding factors.

**Results:**

7418 blood results from 326 patients were included. There was no relationship between serum sodium concentration and estimated glomerular filtration rate at baseline. After multivariable adjustment, a 1 mmol/L increase in baseline serum sodium was associated with a 1.5 mL/min/1.73 m^2^ decline in eGFR during the study period (95% CI 0.9, 2.0). A reduction in eGFR was not associated with significant changes in serum sodium concentration over 6 years.

**Conclusion:**

Higher serum sodium concentration is associated with the progression of CKD, independently of other established risk factors. Conversely, significant alterations in serum sodium concentration do not occur with declining kidney function.

## Introduction

Higher dietary salt consumption in those with chronic kidney disease (CKD) is associated with increased blood pressure and proteinuria, independent risk factors for CKD progression [1]. The mechanisms underlying the adverse effects of salt are incompletely understood, but it has been proposed that small increases in serum sodium concentration are important [2, 3]. In randomised-controlled trials, acute and large alterations in salt intake (ranging from less than 1 g/day to more than 10 g/day) are associated with parallel changes in serum sodium of up to 3 mmol/L [3]. Although changes in extracellular volume also occur, a significant positive correlation between serum sodium and blood pressure exists in both animals and humans when volume status is controlled using dialysis [4, 5]. In addition, there is in vitro evidence that small increases in sodium concentration have a direct effect on the vascular endothelium: experiments have demonstrated stiffening of endothelial cells, damage to the glycocalyx layer and inhibition of nitric oxide release [6, 7]. Furthermore, increased sodium concentration has been shown to induce mRNA expression of many hypertrophy-related factors, including transforming growth factor-beta (TGF-β), that are associated with the progression of CKD [8, 9].

Whilst significant abnormalities in serum sodium, especially hyponatraemia, have been consistently associated with mortality in CKD, the influence of small inter-individual differences has largely been ignored [10–13]. Higher serum sodium concentrations, within the normal range, have recently been reported be a risk factor for the development of incident CKD [14, 15]. However, whether there is a relationship between serum sodium and the progression of established CKD remains unknown. Therefore, we hypothesised that increased serum sodium is a risk factor for renal function decline and carried out a retrospective analysis to investigate this. We also considered the possibility that changes in serum sodium may occur secondary to CKD progression and altered sodium handling by the kidney.

## Methods

### Study design and population

This was a retrospective cohort study of data collected during a 6-year period. The South West Thames Renal Department database (Clinical Vision 5.3) was used to identify individuals under the follow-up of general nephrology services between January 2009 and December 2014. The primary outcome was the change in estimated glomerular filtration rate (eGFR) over the 6-year study period. In order to acutely assess this, the study was separated into three 2-year periods and only included individuals who had had at least three results during each of these periods. Data collected during the first 2-year period was used to determine baseline values. Individuals with a mean eGFR of < 60 mL/min/1.73 m^2^ at baseline were included in the study. Exclusion criteria were as follows: provision of renal replacement therapy during the study period; a diagnosis of congestive cardiac failure, diabetes mellitus (type I or II), or decompensated liver disease prior to the study end date.

### Data collection

Demographic details and laboratory values were extracted from the database and further information was identified manually from clinic letters including cause of CKD, co-morbidity, office blood pressure and medication. Blood test results corresponding to the same date for serum sodium, potassium, creatinine and haematocrit were obtained and an eGFR was calculated for each creatinine value using the CKD-EPI equation [16]. Blood samples were processed at a number of laboratories in the local area, all of which are monitored by the United Kingdom National External Quality Assessment Service (NEQAS) to ensure comparable results. Serum creatinine was measured by enzymatic assay and serum sodium was determined using indirect potentiometry. In order to minimise the effect of acute illness or admission to hospital, blood tests were excluded from the analysis if any of the following applied: the sodium result was missing or significantly abnormal (< 125 mmol/L or > 155 mmol/L); the sample was taken ≤ 7 days apart from another blood test; there was an acute change in creatinine of > 50% from the previous baseline.

Baseline laboratory values were determined for each individual using the mean test result over the first 2 years of the study. Baseline proteinuria was estimated using urine protein:creatinine ratio (PCR) and urine albumin:creatinine ratio (ACR) results: significant proteinuria was defined as a mean PCR of ≥ 50 mg/mmol or a mean ACR of ≥ 30 mg/mmol. Baseline blood pressure was calculated using the mean office measurement from visits during the baseline period. Where multiple measurements were recorded for a single visit, the lowest reading was used to minimise the ‘white-coat’ effect. Blood pressure control was defined as a mean systolic reading of ≤ 140 mmHg and a mean diastolic reading of ≤ 90 mmHg. Finally, prescription data was obtained from nephrology clinic letters during the baseline period, using the most recent available medication list.

### Statistical analysis

Data were analysed using R version 3.2.1 (2015-06-18) with a P value of less than 0.05 being regarded as statistically significant. Changes in eGFR (and serum sodium) during the 6-year study period were determined for each individual using ordinary least squares regression coefficients, with time as the independent variable. Baseline analysis was carried out using univariate linear regression to obtain crude associations between serum sodium and eGFR, as well as other explanatory variables. Differences between two groups were analysed using the unpaired Student’s t-test for normally-distributed variables. Multiple linear regression analysis was used to investigate the relationship between baseline serum sodium and eGFR decline, adjusting for potential confounding factors (age, sex, race, cause of CKD, blood pressure, proteinuria, baseline eGFR, serum potassium, haematocrit and medications). Blood pressure and proteinuria were included as categorical variables because of missing data. The model met the assumptions of normality, linearity and homoscedasticity, and influential outliers were not identified. Finally, linear regression was also used to investigate changes in serum sodium and potassium with eGFR decline.

## Results

### Cohort selection and data completeness

**Fig. 1:**
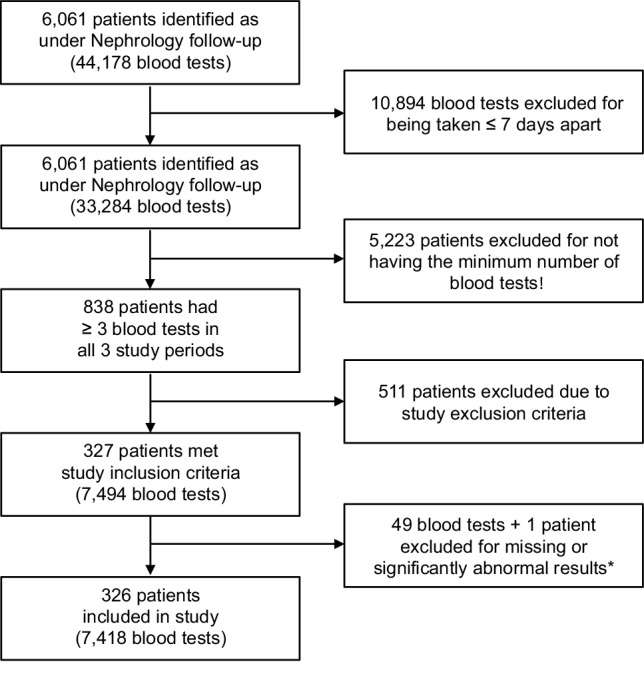
Flowchart demonstrating an overview of the study cohort selection process. *Significantly abnormal result = sodium result of < 125 or > 155 mmol/L, or an acute change in creatinine of > 50% from the previous baseline

Figure 1 demonstrates an overview of the cohort selection process. This resulted in 7418 blood tests being included in the analysis for 326 individuals. Blood tests were collected over a mean period of 2,014 days (SD 132). For each individual, there was a median of 7 (IQR 5–10) blood tests during the baseline period and 13 (IQR 10–19) during the follow-up period. Data was complete for age and sex, in addition to the blood test results. Non-white ethnicity was specified in 30 individuals (9%), and cause of CKD was specified in 147 (45%). Baseline blood pressure readings were obtained for 279 (86%) of the cohort and baseline proteinuria estimations were available for 243 (75%). Prescription data were for available for 278 individuals (85%).

### Baseline associations with serum sodium

**Fig. 2:**
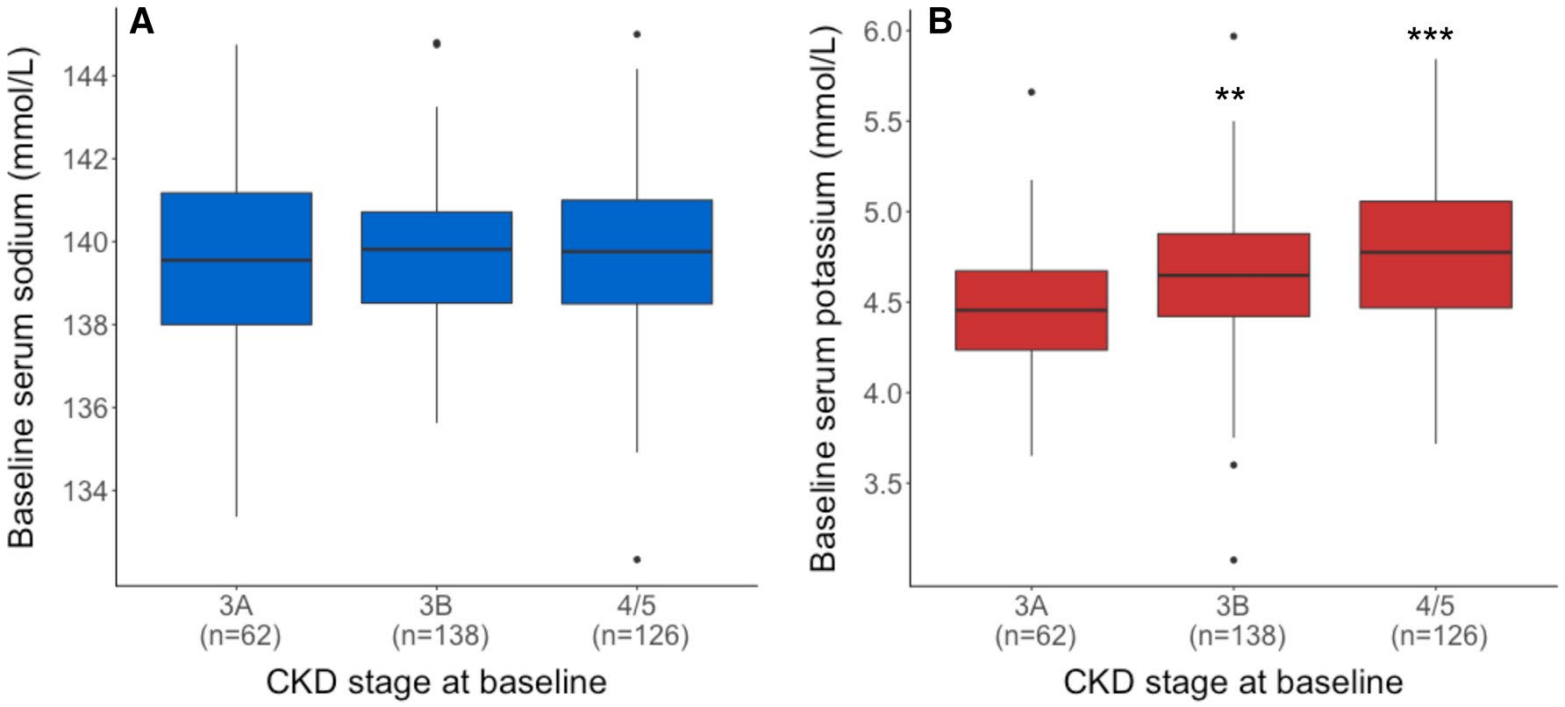
Boxplots demonstrating serum electrolyte concentrations by CKD stage at baseline: **a** sodium; **b** potassium. CKD stage 3A corresponds to an eGFR of between 45 and 59, stage 3B to an eGFR between 30 and 44, and stage 4/5 an eGFR of < 30 mL/min/1.73 m^2^. **P < 0.01; ***P < 0.001 (compared to CKD stage 3A)

The baseline characteristics of the cohort are shown in Table 1. The mean eGFR was  34.2 mL/min/1.73 m^2^ (SD 11.0) and the mean serum sodium concentration was 139.7 mmol/L (SD 2.0). Univariate regression analysis showed a non-significant inverse relationship between eGFR and serum sodium at baseline. In comparison, there was a significant negative association between eGFR and serum potassium (a 1 mmol/L higher serum potassium was associated with a lower eGFR of − 7.1 mL/min/1.73 m^2^; 95% CI 4.3, 9.8). Figure 2 displays the differences in serum sodium and potassium by CKD stage.

**Table 1:** Baseline characteristics of study cohort (n = 326)

Age in years (median, IQR)	70 (15)
Female sex (n, %)	140 (43)
Non-white ethnicity (n, %)	30 (9)
Cause of CKD (n, %)	
Unspecified cause	179 (55)
Glomerulonephritis/vasculitis	61 (19)
Reflux/obstruction	20 (6)
Renovascular/hypertension	32 (10)
Other renal diagnoses	34 (10)
Laboratory results	
Sodium, mmol/L	139.7 ± 2.0
Potassium, mmol/L	4.7 ± 0.4
eGFR, mL/min/1.73 m^2^	34.2 ± 11.0
Haematocrit, %	38.6 ± 4.0
Proteinuria (n, %)	
None or low-level	174 (53)
PCR ≥ 50 or ACR ≥ 30 mg/mmol	69 (21)
Unknown	83 (26)
Blood pressure control (n, %)	
≤ 140/90 mmHg	184 (57)
> 140/90 mmHg	95 (29)
Unknown	47 (14)
Medications (n, %)	
Loop diuretic	56 (17)
Thiazide diuretic	54 (17)
Other diuretic	6 (2)
RAS inhibitor	193 (59)
Other antihypertensive agent	167 (51)
Unknown	48 (15)

Data are shown as mean ± standard deviation unless otherwise specified

*CKD* chronic kidney disease, *eGFR* estimated glomerular filtration rate (CKD-EPI equation), *PCR* urine protein:creatinine ratio, *ACR* urine albumin:creatinine ratio, *RAS* renin:angiotensin system, *IQR* interquartile range

At baseline, a 1 mmol/L higher serum sodium was associated with a higher haematocrit of 0.4% (95% CI 0.2, 0.6). No relationship between serum sodium and blood pressure or proteinuria was demonstrated. The mean serum sodium was lower in individuals prescribed diuretics compared to those not, but the difference was not significant (139.5 and 139.9 mmol/L respectively; P = 0.17). There was also no significant difference in serum sodium observed with the prescription of renin angiotensin system (RAS) inhibitors.

### Relationship between baseline serum sodium and CKD progression

The mean decline in eGFR during the 6-year study period was − 4.2 mL/min/1.73 m^2^ (SD 10.7). On univariate analysis, a 1 mmol/L increase in serum sodium was associated with a − 1.4 mL/min/1.73 m^2^ decline in eGFR during the study (95% CI − 2.0, − 0.8). Table 2 displays the results of the multiple regression model, with change in eGFR during the study period as the dependent variable. The variables included in this model accounted for 27% of the variation in eGFR change. Younger age, male gender, non-white ethnicity, proteinuria and a lower hematocrit at baseline were all significantly associated with a decrease in eGFR. In addition, there was a significant correlation with serum sodium such that a difference of 1 mmol/L at baseline was associated with a decline in eGFR of − 1.5 mL/min/1.73 m^2^ during the study (95% CI − 2.0, − 0.9). Serum potassium and the prescription of diuretics and/or RAS inhibitors were not associated with eGFR loss.

**Table 2:** Multivariate linear regression analysis of factors associated with change in eGFR during the 6-year study period

	*b*	95% CI	P value
Age (years)	0.14	0.05, 0.23	< 0.01
Female sex	3.41	1.10, 5.72	< 0.01
Non-white ethnicity	− 5.33	− 9.13, − 1.53	< 0.01
Cause of CKD			
Unspecified cause	[REF]	[REF]	[REF]
Glomerulonephritis/vasculitis	− 0.57	− 3.74, 2.61	0.73
Reflux/obstruction	− 0.22	− 4.91, 4.46	0.93
Renovascular/hypertension	0.36	− 3.34, 4.07	0.85
Other renal diagnoses	− 2.75	− 6.39, 0.89	0.14
Baseline laboratory results			
Baseline sodium (mmol/L)	− 1.47	− 2.03, − 0.91	< 0.001
Baseline potassium (mmol/L)	− 0.26	− 3.13, 2.61	0.86
eGFR (mL/min/1.73 m^2^)	0.04	− 0.08, 0.16	0.53
Haematocrit (%)	0.42	0.11, 0.74	< 0.01
Proteinuria			
No proteinuria	[REF]	[REF]	[REF]
PCR ≥ 50 or ACR ≥ 30 mg/mmol	− 4.73	− 7.73, − 1.73	< 0.01
Unknown	2.22	− 0.45, 4.88	0.10
Blood pressure control			
≤ 140/90 mmHg	[REF]	[REF]	[REF]
> 140/90 mmHg	0.22	− 2.29, 2.72	0.87
Unknown	− 2.21	− 6.64, 2.23	0.33
Medications			
Loop diuretic	1.29	− 1.89, 4.46	0.43
Thiazide diuretic	0.50	− 2.55, 3.55	0.75
Other diuretic	3.38	− 4.69, 11.44	0.41
RAS inhibitor	− 2.30	− 4.86, 0.27	0.08
Other antihypertensive agent	− 2.17	− 4.63, 0.29	0.08
Unknown	− 1.43	− 6.28, 3.42	0.56

*CKD* chronic kidney disease, *eGFR* estimated glomerular filtration rate (CKD-EPI equation), *PCR* urine protein:creatinine ratio, *ACR* urine albumin:creatinine ratio, *RAS* renin:angiotensin system, *CI* confidence interval

### Changes in serum sodium with eGFR decline

The mean difference in serum sodium concentration between the baseline period and the final 2 years of the study was 0.3 mmol/L (SD 1.5). There was a trend for serum sodium concentration to decrease with greater reductions in eGFR during the study period, but this was not significant (*b* = − 0.02 mmol/L for every − 1 mL/min/1.73 m^2^ reduction in eGFR; 95% CI − 0.04, 0.00). Furthermore, no significant relationship was found in a subgroup analysis of individuals with an eGFR of < 30 mL/min/1.73 m^2^ at baseline (n = 132). In comparison, for the whole cohort, a decrease in eGFR of − 1 mL/min/1.73 m^2^ was associated with a significant increase in serum potassium of 0.02 mmol/L (95% CI 0.01, 0.02).

## Discussion

In this study, there was an association between higher serum sodium concentration and subsequent eGFR decline. After adjustment for confounding variables, a 5 mmol/L increase in baseline serum sodium was associated with a loss in eGFR of − 7.4 mL/min/1.73 m^2^ over 6 years in individuals with established CKD. This finding is interesting in that the association occurs within the normal range of serum sodium and it supports our primary hypothesis that increased serum sodium is a risk factor for CKD progression. It is also notable because adverse outcomes in CKD, in particular mortality, have more frequently been associated with hyponatraemia [10–13]. However, we excluded serum sodium results during acute illness and in those with significant co-morbidity including heart failure. Both may result in lower serum sodium and have a confounding influence on CKD progression, either as a consequence of decompensated disease or the initiation or up-titration of diuretic therapy.

Dietary salt may contribute to higher serum sodium concentrations but there was no association between serum sodium and blood pressure in this investigation. The use of office-based blood pressure readings was a limitation in this respect, but there could be alternative explanations for the relationship between increased serum sodium and eGFR decline. In particular, higher serum sodium could be an indicator of reduced extracellular volume, as evidenced by the significant positive relationship with haematocrit at baseline in our study. Although the effect of serum sodium on CKD progression was independent of this, haematocrit is a crude marker of hydration state and differences in extracellular volume cannot be excluded. However, at present there is limited evidence linking habitual water intake to serum sodium concentration or the progression of CKD [17, 18]: in the recently published CKD Water Intake Trial, increasing water intake did not result in significant alterations in either [18]. It is also interesting to speculate as to the potential role of vasopressin release that occurs with higher sodium concentrations [19]. Following on from animal studies that have implicated a causal role for vasopressin in impaired renal function, there is a growing body of evidence demonstrating an association between copeptin levels (a surrogate marker of vasopressin) and eGFR decline in those with CKD [20, 21].

Finally, we found intra-individual serum sodium to be stable over the 6-year study period, consistent with recent reports of there being significant individuality in serum sodium concentration [22]. We considered the possibility that the advancement of CKD could affect the ability of the kidneys to regulate salt and water balance, particularly as a greater prevalence of hypernatraemia has been reported with progressive stages of CKD [10]. However, this study did not find that declining kidney function was associated with a significant change in serum sodium. There were also no significant alterations in serum sodium in a subgroup of individuals with CKD stages 4–5 at baseline, despite the fact that the capacity to produce concentrated urine declines with glomerular filtration rates less than 30 mL/min/1.73 m^2^ [23]. Whilst it is possible that the study was underpowered to detect such a change, this suggests that our primary findings are not explained by an effect of CKD progression on serum sodium.

### Limitations

This study has a number of important limitations, including a relatively small cohort: the selective design was necessary in order to detect small changes in serum sodium in the presence of confounding variables. However, together with the retrospective design, this may have resulted in selection bias such that those with stable disease or those with rapidly progressive disease were excluded. Linear regression coefficients were used to estimate eGFR decline during the study but non-linear changes over time may limit the accuracy of this method. Furthermore, the data was collected routinely rather than for research purposes and this has implications for completeness and accuracy. In particular, there are limitations to using office blood pressure readings and prescription data may be unreliable due to dose alterations and compliance issues. Finally, the confounding effects of unmeasured factors influencing CKD progression cannot be excluded.

## Conclusion

To our knowledge, this study is the first to demonstrate an association between higher serum sodium concentration and subsequent eGFR decline in people with established CKD. The observational nature of the study does not ascertain causality but, together with evidence that dietary salt intake increases serum sodium, this data is consistent with guidelines advocating the importance of salt restriction in this group. However, inter-individual differences in serum sodium concentration are not attributable to differences in salt intake alone. Further studies would be of value to further investigate the link between serum sodium, dietary salt, water intake and vasopressin activity, as well as their relationships with long-term health outcomes in those with CKD.
